# Comparison of Mannitol, Water, and Iodine-Based Oral Contrast in the Evaluation of the Bowel by Multi-Detector Computed Tomography

**DOI:** 10.7759/cureus.24316

**Published:** 2022-04-20

**Authors:** Sai Soumya Thati, Rachegowda Nagegowda, Anil K Sakalecha, Shivaprasad G Savagave, Divya T Patil

**Affiliations:** 1 Radiology, Sri Devaraj Urs Medical College, Kolar, IND; 2 Radiodiagnosis, Sri Devaraj Urs Medical College, Kolar, IND

**Keywords:** small bowel, positive oral contrast, mural characteristics, mannitol, large bowel, computed tomography, bowel distension

## Abstract

Background and objectives

To perform contrast-enhanced computed tomography (CECT) of the abdomen with water, mannitol, or iodinated positive contrast as an oral contrast agent, and compare the distension and enhancement pattern of the bowel.

Methods

This was a prospective observational study conducted on 90 patients over a period of 12 months who were referred for CECT abdomen. Patients were randomly divided into three groups (30 each) and were given water, mannitol, or positive oral contrast before the CECT study. Quantitative and qualitative analysis of the bowel for distension, mural fold pattern, and enhancement was analyzed at various anatomical levels. A qualitative examination of bowel loops was done in the three groups by using a continuous 4-point scale.

Results

The mean distension at the duodenum was 1.89 ± 0.33 cm (mean ± SD) with water, 2.28 ± 0.36 cm with mannitol, and 2.01 ± 0.33 cm with positive oral contrast. Overall, maximum luminal distension was seen at the level of the duodenum, followed by the jejunum across all the groups. Bowel characteristics were far superior in the mannitol group compared to water and positive oral contrast at all anatomical levels.

Conclusion

Small bowel distension was excellent with mannitol, followed by positive oral contrast, and least with water. Mural characteristics and enhancement patterns were better with mannitol as compared with water and with positive oral contrast.

## Introduction

The small bowel is a challenging area for the surgeon due to its long length and vague symptomatology, often making the radiologist an indispensable part of the diagnostic team [1]. Due to bowel gas artifacts, ultrasound is less sensitive for evaluating the bowel. Computed tomography (CT) has good spatial/contrast resolution and is considered a better modality for the evaluation of bowel pathologies [2,3].

Since radiologists assume the prime responsibility for the diagnostic evaluation of the small bowel, it is essential that the methods capable of accurately demonstrating small bowel morphology are appropriately employed [4]. Barium investigations are helpful in detecting intraluminal pathologies but are often non-specific with low diagnostic yield [2,5]. Small bowel capsule endoscopy is a recent imaging modality for small intestinal pathologies. But due to its inability to evaluate extramural pathologies, its application is restricted [6]. Conventional abdominal CT depicts extramural pathologies but usually overlooks intraluminal and intramural disease [7]. Multi-detector computed tomography (MDCT) with optimal enteral contrast agent has overcome the above-mentioned limitations and has revolutionized the imaging of bowel [8-10].

There is a paucity of data on what constitutes an ideal oral contrast agent. Due to its high osmolarity, mannitol can be used as an oral contrast agent, and it is hypothesized to provide better distension of bowel loops [11,12]. The objective of this study was to demonstrate that bowel distension and enhancement patterns on MDCT are better with mannitol in comparison with water and iodinated oral contrast. This study was performed using a basic 16-slice CT machine, which is widely available even in rural India and makes it unique compared to similar studies done thus far.

## Materials and methods

This was a hospital-based observational study performed over a period of 12 months on 90 patients referred for contrast-enhanced computed tomography (CECT) abdomen to the Department of Radiodiagnosis at R. L. Jalappa Hospital, attached to Sri Devaraj Urs Medical College, Kolar, Karnataka, after obtaining approval from the Institutional Ethics Committee (IEC). Informed consent was obtained from individuals for their willingness to participate in the study. The following method of sample size calculation was used in this study.

Sample size calculation 

Keeping the minimum mean difference of bowel distension between the groups as 0.5 at the level of jejunum with a standard deviation of 0.7 and 0.4, at 95% confidence interval and 80% power [1], formula used,

$N=2*S_{p}^{2}*\left [ Z_{1-\alpha /2}+Z_{1-\beta } \right ]^{2}/\mu _{d}^{2}$

$S_{p}^{2}= \left [ S_{1}^{2} +S_{2}^{2}\right ]/2$

where *S*_1 _is the standard deviation of the first group, *S*_2 _is the standard deviation of the second group, *µ*_d_ is the mean difference between samples, *α* is the significance level, and 1-β: is the power. Based on these assumptions, the minimum sample size works out to be 3 * 20 = 60. However, the actual sample size was 90 patients.

Inclusion criteria

Patients who were referred to the Department of Radio-diagnosis for CECT abdomen in view of non-gastro-intestinal pathologies and individuals over 18 years of age were included in this study.

Exclusion criteria

Patients who are allergic or have a past history of allergy to contrast, patients with deranged renal function tests, and pregnant women were excluded from the study.

Data collection and CT protocol

Individuals were randomly divided into three groups and were given either 1500 mL of plain water (group I), 3% mannitol (group II), or iodinated oral contrast (15 mL in 1500 mL) (group III), 45 minutes prior to the CT scan. They were instructed to consume around 250 ml every 10 minutes for a period of 45-50 minutes. A plain CT was performed with the patient in the supine position using the breath-holding technique by a 16-slice Siemens® Somatom Emotion® scanner. Contrast-enhanced CT was performed with a multiphase study, which included arterial and venous sequences. A contrast agent, Iohexol 300 (Ultravist®), was injected into all patients intravenously at a rate of 3.5 to 4 mL/s using a pressure injector.

Image assessment

The images were transferred to a work station (Myrian® or Osirix®), where they were reported by two radiologists who were blinded to the neutral luminal contrast agents, i.e., mannitol and water. A quantitative and qualitative evaluation of bowel distension was done using the images. Axial, coronal, and sagittal reformatted images of the venous phase were chosen for evaluation. Bowel distension was evaluated at various levels: duodenum at one level, jejunum, ileum at two levels, and ileo-caecal junction. The measurements were taken in axial and coronal planes, taking the outer to outer diameter into account. The duodenum was measured in the second part. The jejunum was measured at two points (JI and J2) at the level of the superior mesenteric artery (SMA), medial and lateral to it. The ileum was measured at the bifurcation of the iliac vessels and in the right iliac fossa (I1 and I2). The values and findings of both radiologists were compared, and mean values were acquired at each anatomical level.

Analysis of the bowel for distension, mural fold pattern, enhancement, and image quality was done by diameter measurement and point scale system at various anatomical levels, i.e., duodenum, jejunum, and ileum. A qualitative examination of bowel loops was done in the three groups by using a continuous 4-point scale: 0-3, fair to excellent, percentage of small bowel loops showing adequate distension or homogeneity of luminal contents or fold visibility [2].

Score 0: Fair (<25%)

Score 1: Good (25-50%)

Score 2: Very good (50-75 %)

Score 3: Excellent (>75%)

Additionally, the visibility of mural fold characteristics across all the groups was measured using a semiquantitative score. In this system, a score of 0 was given if the bowel loops were partially or completely collapsed with poor mural fold visibility; a score of 1 was given if mural fold visibility was good, and a score of 2 was given when there was excellent mural fold visibility. A qualitative assessment of large bowel distension was performed on the ascending, transverse, and descending colons. Features like haustral visibility and the degree of large bowel distension were graded as good, average, and poor based on subjective assessment.

Statistical analysis

Data were entered into a Microsoft Excel datasheet (Microsoft® Corp., Redmond, WA) and were analyzed using SPSS 22 version (IBM Corp., Armonk, NY) and OpenEpi software. Categorical data were represented in the form of frequencies and proportions. The Chi-square test was used as a test of significance for qualitative data and the analysis of variance (ANOVA) test was used as a test of significance for quantitative data. A P-value of <0.05 was considered statistically significant.

## Results

Age and gender distribution

We included 90 patients in our study who were randomly distributed into three groups (n = 30 in each group). There was no significant difference in the age group of patients in either of the groups (P = 0.18; not significant) (Table 1). The total mean age was 46.9 ± 14 years, with a range of 18-75 years. In a total of 90 cases, there were 45 males and 45 females, with no significant difference in gender distribution across the three groups.

Quantitative evaluation

**Table 1:** Age and gender distribution of cases. NS = not significant; SD: standard deviation

	Water	Mannitol	Positive contrast	P-value
Age (in years) (mean ± SD)	51 ± 11.86	44.43 ± 14.92	45.5 ± 16.87	0.18; NS
Gender				
Males	15	12	18	NS
Females	15	18	12	
Total	30	30	30	

**Figure 1:**
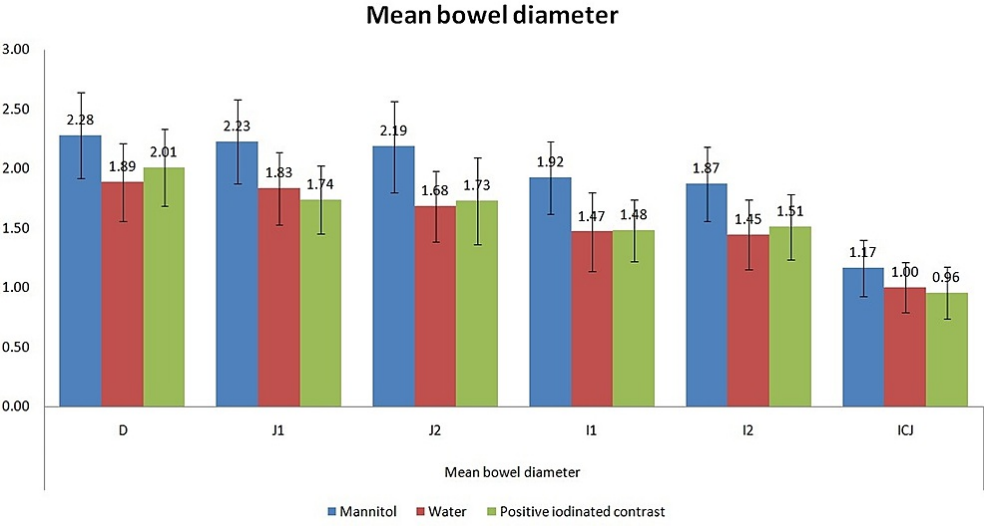
Mean bowel diameter across various groups.

The mean distension at the duodenum was 1.89 cm with water, 2.28 cm with mannitol, and 2.01 cm with positive oral contrast. Overall, maximum luminal distension was seen at the level of the duodenum, followed by the J1 site (at the level of the origin of SMA) across all the groups. Among all the groups, maximal luminal distension was seen in the mannitol group, irrespective of the site (Table 2; Figure 1). There was significantly better luminal distension with mannitol when compared with water (P<0.001 at all sites except ileocecal junction [ICJ]; P = 0.006 at ICJ) or positive iodinated contrast (P<0.001 across all sites). The luminal distension provided by water and positive iodinated contrast was similar, with no statistically significant difference among these groups at all sites (Table 2).

**Table 2:** Mean bowel diameter at different locations across all groups. D: duodenum; J1, J2: jejunal sites; I1, I2: ileal sites; ICJ: ileocecal junction, PIC: positive iodinated contrast.

Mean bowel diameter (in cm)					P-value (mannitol vs water)	P-value (mannitol vs PIC)	P-value (water vs PIC)
Type of contrast		Water	Mannitol	PIC			
Bowel segment	D	1.89 ± 0.33	2.28 ± 0.36	2.01 ± 0.33	<0.001	<0.001	0.15
	J1	1.84 ± 0.31	2.23 ± 0.36	1.74 ± 0.29	<0.001	<0.001	0.23
	J2	1.69 ± 0.3	2.19 ± 0.39	1.73 ± 0.37	<0.001	<0.001	0.62
	I1	1.47 ± 0.34	1.93 ± 0.31	1.48 ± 0.26	<0.001	<0.001	0.89
	I2	1.45 ± 0.3	1.87 ± 0.32	1.51 ± 0.28	<0.001	<0.001	0.4
	ICJ	1.01 ± 0.22	1.17 ± 0.24	0.96 ± 0.22	0.006	<0.001	0.41

Qualitative evaluation

**Figure 2:**
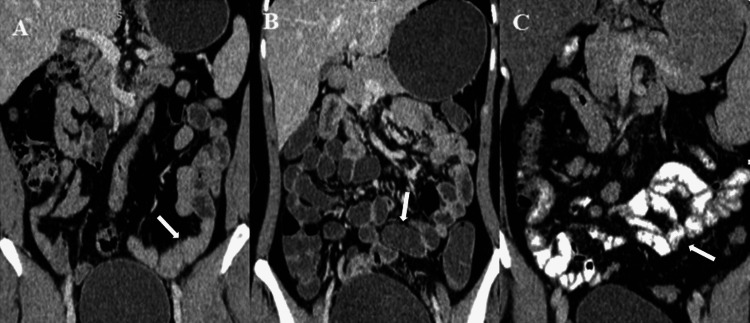
(A-C) Coronal reformatted CECT image showing bowel distension with oral plain water (A), bowel distension with mannitol (B), and bowel distension with positive oral contrast (C). Note the excellent bowel distension with mannitol when compared with water and positive iodinated contrast. CECT: contrast-enhanced computed tomography.

**Figure 3:**
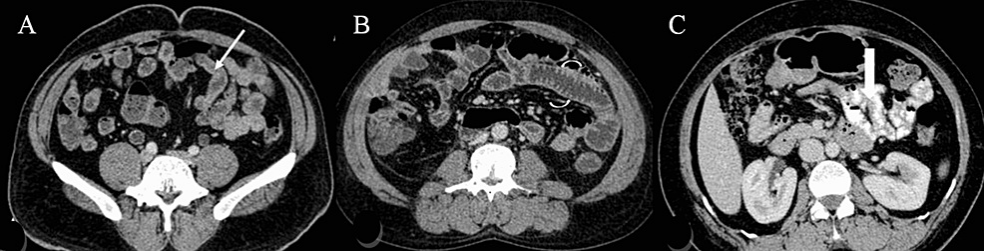
(A-C) Axial CECT abdomen in patient who was given water showing poor mural features (thin white arrow in A), in patient who was given mannitol showing good mural fold visualization (curved white arrows in B) and patient who was given positive iodinated contrast (C), which shows suboptimal mural fold visualization (thick white arrow).

**Figure 4:**
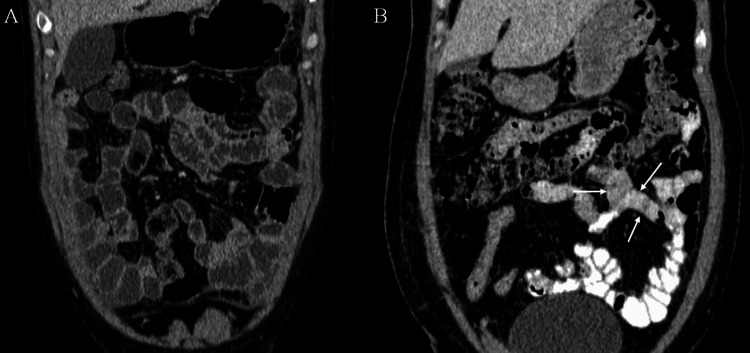
Coronal CECT Abdomen in patients given mannitol (A) and positive oral contrast (B). Note the good mural fold visualization in patient who was given neutral oral contrast like mannitol (A) and suboptimal visualization of mural folds in patient with positive oral contrast (white arrows in B).

It can be seen from Table 3 that 60% of patients using mannitol showed excellent bowel distension or homogeneity of luminal contents or fold visibility, and only 10% of patients who had water as an oral contrast showed excellent distension. The degree of bowel distension or homogeneity of luminal contents/mural fold pattern was significantly better with mannitol when compared with water or positive iodinated contrast (P<0.001), and the degree of bowel distension or homogeneity of luminal contents was significantly better with positive iodinated contrast when compared with water (P = 0.01). Overall, mannitol showed better bowel distension or homogeneity of luminal contents/mural fold pattern, followed by positive iodinated contrast and water on imaging (Figures 2-4).

**Table 3:** Qualitative evaluation of small bowel loops across all groups. P-value = significant across all groups (<0.001). P-value is significant between mannitol and water (P<0.001), between mannitol and positive oral contrast (P= 0.03), and between water and positive oral contrast (P = 0.01). %: percentage.

	Water		Mannitol		Positive iodinated contrast	
Grade	Count	%	Count	%	Count	%
0	8	27	1	3	2	7
1	14	47	3	10	8	27
2	5	17	8	27	13	43
3	3	10	18	60	7	23
Total	30		30		30	

Mural fold characterization

Table 4 shows the mural fold pattern across the three groups. Mannitol showed excellent mural fold visibility in more than 50% of cases (n = 17; 56.67%). The degree of mural fold visibility was significantly better with mannitol when compared with water or positive iodinated contrast (P<0.001). In contrast, water showed poor mural fold visibility in more than 60% of cases. Positive iodinated contrast showed poor mural fold visibility in 90% of the cases. Water showed better mural fold visibility when compared with positive iodinated contrast (P<0.05). Overall, mannitol showed the best mural pattern, followed by water and positive iodinated contrast.

**Table 4:** Comparison of mural characteristics of bowel. P<0.001 for mannitol versus positive oral contrast and water. P<0.001 for water and mannitol and for mannitol and positive oral contrast; P<0.05 for water and positive oral contrast.

	Water		Mannitol		Positive iodinated contrast	
Score	Patients	%	Patients	%	Patients	%
Score 0	19	63.3	2	6.66	27	90.0
Score 1	8	26.6	11	36.67	3	10.0
Score 2	3	10.0	17	56.67	0	0
Total	30		30		30	

Large bowel evaluation

**Figure 5:**
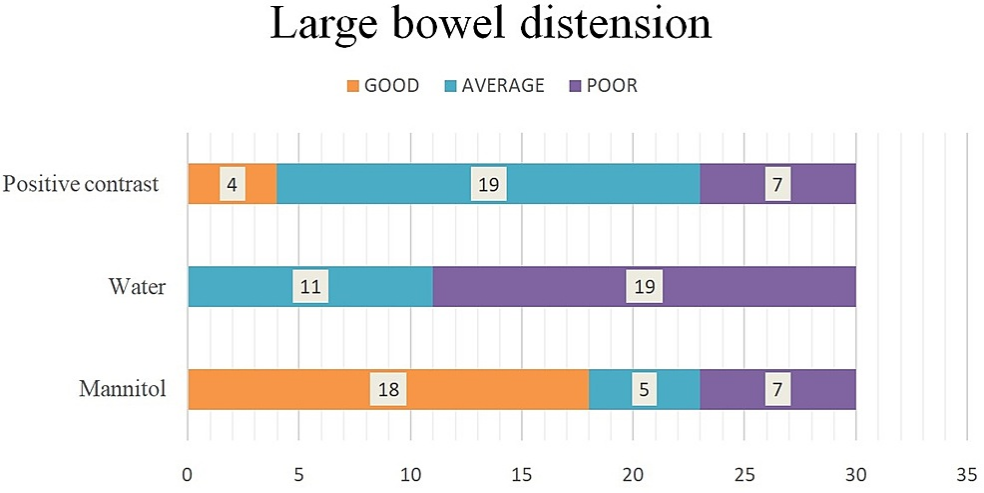
Large bowel distension with water, mannitol, and positive iodinated contrast.

Mannitol showed good large bowel distension in 60% of cases, with an average distension in five patients (16.67%) and poor distension in seven patients (23.33%). None of the patients who had water showed good large bowel distension. Patients who received positive iodinated contrast showed good large bowel distension only in four cases (13.34%) (Figure 5). The degree of large bowel distension was significantly better with mannitol when compared with water and positive iodinated contrast (P<0.001). Similarly, positive iodinated contrast showed significantly better large bowel distension when compared with water (P<0.05).

## Discussion

The high resolution provided by MDCT images has changed the approach to small bowel diseases. It has excellent spatial and contrast resolution, which helps in excellent visualization of bowel loops [1]. Its ability to visualize both the bowel wall and the bowel lumen provided additional anatomical information that was not provided by barium studies. An ideal endoluminal contrast agent should provide optimal luminal distension, provide adequate mural details, and should be of low attenuation [2]. There are three types of oral contrast agents, i.e., positive, negative, and neutral oral contrast agents [13]. Neutral agents are always preferred over positive or negative oral agents as they cause homogenous distension of the bowel loops. Zheng et al. and Minordi et al. concluded that among neutral agents, polyethylene glycol was the least preferred agent [14,15].

Berther et al. reported a nearly equal male/female ratio in their study, which is similar to our study (101:99) [16]. In our experience, we have observed that for adequate bowel distension, the use of at least 1500 mL of endoluminal contrast is necessary. Various studies have used different amounts of endoluminal contrast ranging from 1000 mL to 1800 mL [1,2,5,17]. Most of the studies, however, used 1500 mL of endoluminal contrast [1,2,18].

We performed a contrast study after about 45 minutes of the initiation of oral contrast. Our protocol is supported by Meindl et al., who compared the luminal distension with the short protocol of oral endoluminal contrast (1000 mL for 60 minutes) and the prolonged protocol (2000 mL for 120 minutes). Additionally, the prolonged protocol is recommended while evaluating the ileocecal junction and large bowel [19].

The high osmolarity of an endoluminal contrast can be considered to be the single most important factor governing bowel distension [2]. Mannitol is an osmotic agent, which is cheap, easily available, and hence was considered as a potential replacement for water in our study [6,7]. Water mixed with mannitol and the physiologic secretions of the upper GI tract has a similar neutral fluid attenuation, which provides for homogeneous images on CT [2,5]. Additionally, it augments its ability to provide consistent bowel distension [6]. Water has been shown to be rapidly absorbed by the bowel mucosa and therefore is known to cause suboptimal bowel distension in the distal small bowel loops [2,20]. 

In our study, there was significantly better luminal distension with mannitol when compared with water (P<0.001 at all sites except ICJ; P = 0.006 at ICJ) and positive iodinated contrast (P<0.001 across all sites). The mean luminal measurements at the ileum and ileocecal junction in our study are similar to those observed by Wang et al., who reported a mean ileal diameter of 2.15 ± 1.3 cm (mean ± SD) each at the ileum and ileocecal junction with mannitol, and 1.01 ± 0.05 cm (mean ± SD), 0.99 ± 0.06 cm (mean ± SD) at the ileum and ileocecal junction, respectively, with water [6].

The degree of bowel distension or homogeneity of luminal contents was significantly better with mannitol (P<0.001). Water was the least effective agent in achieving adequate bowel distension or homogeneity of luminal contents. Additionally, water showed better mural fold visibility when compared with positive iodinated contrast (P<0.05). Overall, mannitol showed better bowel distension or homogeneity of luminal contents and mural fold visibility when compared with water and positive iodinated contrast.

Due to its osmotic property, better luminal distension and homogenous attenuation were achieved by mannitol. The rapid absorption of water by the bowel mucosa can be attributed to its poor performance in providing adequate bowel distension or homogeneity of luminal contents/fold visibility [2,21]. The osmolality of mannitol is higher than positive iodinated contrast, therefore providing better results in demonstrating bowel distension or homogeneity of luminal contents/fold visibility [1].

Factors responsible for poor bowel wall demonstration with positive contrast are high density of contrast, potentially causing artifacts, and partial volume averaging (less likely with iodinated contrast) [1,2]. Multiple artifacts may also be present due to differential attenuation resulting in increased concentration in some areas [22]. This finding is of paramount importance in diagnosing inflammatory and ischemic conditions of the bowel where mural characterization is important [2,23].

In our study, the visualization of the distal ileum and ileocaecal junction was excellent with mannitol and poor with water, a finding supported by Kaireit et al. [24]. While the use of water may not cause adequate distension of these areas, the use of positive iodinated contrast can obscure the mural enhancement features, which makes it difficult to accurately assess these regions [1,2]. Mannitol overcomes these limitations and therefore can be considered superior in the evaluation of ileocaecal junction pathologies [25].

In cases of perforation, fistula, or suspected anastomotic leak, neutral oral contrast agents do not provide any additional information. Also, certain cystic lesions of the mesentery or the pelvis cannot be differentiated from bowel lesions when neutral oral contrast is given. Hence, in such cases, oral positive iodinated contrast may be useful [26].

Limitations

This study has a few limitations as only a small group of patients could be evaluated. A larger sample size would have given very conclusive evidence of the hypothesis.

## Conclusions

In our study, the bowel distension was compared after the administration of three oral contrast agents, namely, mannitol, positive oral contrast, and water. Quantitative-qualitative evaluation of the bowel on computed tomography showed that mannitol demonstrated much superior, homogeneous bowel distension and mural characteristics as compared to the rest of the oral contrast agents.

We recommend the use of mannitol on a routine basis for CECT abdomen studies irrespective of the indication, as it is easily available, non-toxic, and superior to both water and positive oral contrast in the evaluation of the bowel even on a basic 16-slice computed tomography.
